# The comparison of airway ultrasonography and continuous waveform capnography to confirm endotracheal tube placement in cardiac arrest patients: prospective observational study

**DOI:** 10.1186/2036-7902-4-S1-A6

**Published:** 2012-12-18

**Authors:** Jong Kab Noh, Young Soon Cho

**Affiliations:** 1 Department of Emergency Medicine, College of Medicine, Soonchunhyang University, Korea

## Background

Ultrasound is a common examination tool in many emergency department and intensive care unit. Several studies have provided promising results of the use of ultrasound for the confirmation of endotracheal tube placement

## Objective

This study aimed to assess the accuracy and timeliness of using tracheal ultrasound to examine endotracheal tube placement in cardiac arrest patients.

## Patients and methods

This was a prospective, observational study, conducted at the emergency department of a university teaching hospital. Patients received emergency intubation due to cardiac arrest. Airway ultrasonography was performed during emergency intubation with the transducer placed transversely at the trachea over the suprasternal notch. Quantitative waveform capnography was used as the criterion standard for confirmation of tracheal intubation. The main outcome was the timeliness between the airway ultrasonography and the capnography.

## Results

A total of 16 patients and 19 intubations were included in the analysis. The endotracheal tube was placed in the trachea in 16 intubations and in the esophagus in 3 intubations. The overall sensitivity and specificity of ultrasound to confirm tracheal intubation was 100% respectively. Capnography application times after intubation were 17.5 (10.0~32.5) seconds. The Capnograpny confirmation times after application were 30 (10~120) seconds. Ultrasound confirmation times of endotracheal tube placement after application were 5 (4~5) seconds.

## Conclusion

Ultrasound confirmation was very fast, accurate and not affected by pulmonary blood flow. Ultrasound confirmation of endotracheal tube placement is more useful in emergency department.
